# Merging the occurrence possibility into gene co-expression network deciphers the importance of exogenous 2-oxoglutarate in improving the growth of rice seedlings under thiocyanate stress

**DOI:** 10.3389/fpls.2023.1086098

**Published:** 2023-02-23

**Authors:** Yu-Xi Feng, Li Yang, Yu-Juan Lin, Ying Song, Xiao-Zhang Yu

**Affiliations:** College of Environmental Science and Engineering, Guilin University of Technology, Guilin, China

**Keywords:** rice, thiocyanate, 2-OG, carbon metabolism, nitrogen metabolism

## Abstract

Thiocyanate (SCN^−^) can find its way into cultivated fields, which might hamper the harmony in carbon and nitrogen metabolism (CNM) of plants, ebbing their quality and productivity. In the current study, we investigated the role of the exogenous application of 2-oxoglutarate (2-OG) in maintaining homeostasis of CNM in rice seedlings under SCN^−^ stress. Results showed that SCN^−^ exposure significantly repressed the gene expression and activities of CNM-related enzymes (e.g., phosphoenolpyruvate carboxylase, NADP-dependent isocitrate dehydrogenases, and isocitrate dehydrogenases) in rice seedlings, thereby reducing their relative growth rate (RGR). Exogenous application of 2-OG effectively mitigated the toxic effects of SCN^−^ on rice seedlings, judged by the aforementioned parameters. The co-expression network analysis showed that genes activated in CNM pathways were categorized into four modules (Modules 1–4). In order to identify the key module activated in CNM in rice seedlings exposed to SCN^−^, the results from real-time quantitative PCR (RT-qPCR) tests were used to calculate the possibility of the occurrence of genes grouped in four different modules. Notably, Module 3 showed the highest occurrence probability, which is mainly related to N metabolism and 2-OG synthesis. We can conclude that exogenous application of 2-OG can modify the imbalance of CNM caused by SCN^−^ exposure through regulating N metabolism and 2-OG synthesis in rice seedlings.

## 1 Introduction

Carbon (C) and nitrogen (N) are the two primary nutrient species, and their adequate supply and dynamic balance of both elements should be essential for regulating cellular functions during plant growth and development (Zheng, 2009; Naseeruddin et al., 2018). It is well known that C-rich biomolecules (e.g., sucrose, glucose, and fructose) provide the majority of energy and C-skeletons for ammonium (NH_4_^+^) assimilation, while N-containing compounds are parts of organic (e.g., amino acids and proteins) and simple inorganic compounds (e.g., nitrate [NO_3_^−^] and NH_4_^+^), which can be synthesized through the incorporation of NH_4_^+^ into the C-skeletons (Zheng, 2009). At the enzymatic level, nitrate reductase (NR), glutamine synthetase (GS), sucrose-phosphate synthase (SPS), trehalose-6-phosphate synthase (TPS), and glutamyl tRNA synthetase (ERS) play a dominant role in regulating the carbon and nitrogen metabolism (CNM) in plants (Coruzzi and Zhou, 2001). However, various environmental stimuli, such as pollutants, drought, salinity, fertilization, and extreme temperature, can influence and destabilize CNM-associated enzymes, thereby weakening the yield and quality of crops (Xin et al., 2019; Alves et al., 2021; Guo et al., 2021).

Thiocyanate (SCN^−^), being part and parcel of many industrial activities (e.g., manufacturing of chemical insecticide and herbicide, production of thiourea, metal separation, and gold mining), is continuously marking its imprint in a clean environment (Yu and Zhang, 2013). Notably, gold ore processing generates a large amount of SCN^−^ because of the lixiviant cyanide complexed with the reduced sulfur species in the gold-bearing ore (Gao et al., 2022). Even mine waste is treated before being discharged, with the aim to convert cyanide into SCN^−^ (Gould et al., 2012). Different governing bodies have issued standards regarding the discharge of cyanide-rich effluent in the environment considering its environmental risk and health hazard (Mudder and Botz, 2004); however, discharge of SCN^−^ in effluent has not been restricted by standards, ultimately raising SCN^−^ level in the effluent (Gould et al., 2012). Indeed, the levels of SCN^−^ at 1,000 mg SCN/L were detected in gold tailings wastewaters (Gao et al., 2022). Persistence of higher levels of SCN^−^ in soils, sediments, rivers, and aquatic biota in nearby areas of gold mines has been observed, which eventually makes its entry into the food chain and poses a threat to all living organisms (Bhunia et al., 2000; Yu and Zhang, 2013; Sun et al., 2018; Lin et al., 2020). Indeed, accumulation of SCN^−^ in plants can cause serious damage to plant growth and development by decreasing nutrient balance and transpiration, degrading photosynthetic pigments, changing the free amino acid composition, and inhibiting the activities of antioxidant enzymes (Hansson et al., 2008; Yu and Zhang, 2013). Our previous studies at the physio-biochemical and molecular levels also indicated that SCN^−^ exposure is able to result in the dysfunction of chloroplast (Yang et al., 2021). These studies suggested that the negative effects of SCN^−^ exposure on the CNM in rice seedlings are detectable.

In recent years, the application of plant growth regulators has been suggested to curtail the negative impact imposed by various abiotic factors (Yang et al., 2021). It is evident that 2-oxoglutarate (2-OG) is a key organic acid involved in the processes of CNM in plants (Yue et al., 2018; Ji et al., 2020). Specifically, the GS initially converts NH_4_^+^ into glutamine in an ATP-dependent reaction; afterward, the glutamate synthase (GOGAT) catalyzes the conversion of glutamine and 2-OG into two molecules of glutamate. Clearly, there is a mandatory interaction between N metabolism and C metabolism (Gálvez et al., 1999). In addition, exogenous application of 2-OG can enhance the activities of phosphoenolpyruvate carboxylase (PEPC), GS, and NADP-dependent isocitrate dehydrogenases (NADP-ICDH) in roots of rice (Yuan et al., 2007). Feeding of exogenous 2-OG can also improve the transcripts of N metabolism-related genes in plants (Araújo et al., 2014). These studies suggested the positive feedback of exogenous 2-OG on the CNM in plants. To date, little is known about the role of exogenous 2-OG in regulating the imbalance of CNM induced by SCN^−^ in plants.

Rice (*Oryza sativa* L.) is one of the most important staple food crops worldwide, especially in eastern Asia countries (Mostofa et al., 2014). Nowadays, agricultural crops suffer from various environmental issues. The SCN^−^ is a typical N-containing pollutant that can be assimilated by rice plants. Therefore, in the present study, we hypothesized that SCN^−^ can disturb the CNM in rice plants during the detoxification of exogenous SCN^−^, while the application of exogenous 2-OG can maintain homeostasis of CNM in rice seedlings in response to SCN^−^ exposure. To prove this hypothesis, the following works were performed: 1) we estimated the relative growth rate and percentage of carbon/nitrogen of rice seedlings under SCN^−^ exposure with or without exogenous 2-OG, 2) we analyzed the effects of exogenous 2-OG on CNM-related genes and enzymes under SCN^−^ stress, and 3) we clarified the strategies of exogenous 2-OG to regulate the imbalance of CNM in rice plants under SCN^−^ exposure by merging the occurrence possibility into a co-expression module analysis. Overall, this study provides new evidence to expand our understanding of exogenous 2-OG to regulate the imbalance of CNM in rice plants during SCN^−^ exposure.

## 2 Methods and materials

### 2.1 Plant growth and experiment design

The seeds of a regular medium-maturing indica rice (*O. sativa* L. XZX 45) were sowed in river sand after being soaked in distilled water for 24 h and then moved inside an artificial climate chamber with a controlled temperature of 25°C ± 0.5°C at a relative humidity of 60% ± 2% Zhang et al., 2022. The rice seedlings were irrigated daily with a modified 8692 nutrient solution, which was described in our previous work (Yang et al., 2021). The modified 8692 nutrient solution with KNO_3_ (39.5 mg N/L) was used. After 16-day growth, rice seedlings of similar size were collected and incubated in a MES-Tris solution (pH = 6.0) for 4 h to remove additional ions from the root surface and the apparent free space. These pretreated seedlings were transferred into a nutrient solution spiked with SCN^−^ and utilized in subsequent experiments. Two treatment series were conducted:

(1) SCN^−^ treatments: SCN^−^ spiked solutions at concentrations of 0 (control 1), 24.0, 96.0, and 300.0 mg SCN/L. Control 1 refers to the nutrient solution without SCN^−^ and exogenous 2-OG.
(2) “SCN^−^ + 2-OG” treatments: seedlings were pretreated with a 2-OG solution at a concentration of 4 mmol/L for 4 h (Fritz et al., 2006), and then seedlings were exposed to SCN^−^ solution at 0 (control 2), 24.0, 96.0, and 300.0 mg SCN/L. Control 2 refers to the nutrient solution without SCN^−^, but with exogenous 2-OG.

Exposure concentrations of SCN^−^ used were based on three different effective concentrations (ECs), i.e., EC_20_, EC_50_, and EC_75_, referring to the 20%, 50%, and 75% inhibition of relative growth rates of rice seedlings, respectively (Lin et al., 2020). All seedlings were placed in the plant growth chamber for a 3-day exposure. Potassium thiocyanate (KSCN) of analytical grade purity with 98.5% purity was purchased from Sinopharm Chemical Reagent Co., Ltd. (Shanghai, China). α-Ketoglutaric acid (2-OG) of analytical grade purity with 98.0% purity was obtained from Shanghai Macklin Biochemical Co., Ltd. (Shanghai, China). To minimize water loss and prevent algae growth, each flask was covered with aluminum foil. Each treatment concentration was performed with four independent replicates.

### 2.2 Analysis of growth parameter

The relative growth rate (RGR) is one of the most crucial parameters to reflect the performance of plants under various stresses (Lin et al., 2020). The RGR (%) was calculated using the biomass change of young seedlings during SCN^−^ exposure, as follows:


, (1) $$RGR=\frac{W_{\left(F\right)}-W_{\left(I\right)}}{W_{\left(I\right)}}\times 100\%$$

where *W*_(I)_ and *W*_(F)_ are the initial and final fresh weights of rice seedlings, respectively.

### 2.3 Measurement of total C and N contents in rice tissues

After exposure to the SCN^−^ solution for 3 days, rice seedlings were harvested and separated into roots and shoots. After being washed with double-distilled water, plant materials were oven dried at 90°C for 48 h and weighed. Then, 0.010 g of oven-dried plant materials was grained into fine powder. Total C and N (%) were measured by a vario elemental analyzer (vario EL) (Brown et al., 2007).

### 2.4 Measurements of activities of CNM-related enzymes in rice tissues

Activities of enzymes related to C metabolism, including PEPC (Osuna et al., 1996),ERS (Ratinaud et al., 1983), TPS (Goddijn et al., 1997), and SPS (Feng et al., 2019) in rice tissues were assayed (detailed information is shown in **Supplementary material M1**).

Activities of enzymes activated in N metabolism, namely, NR (Ahanger et al., 2021), nitrite reductase (NiR) (Lin et al., 2022a), and GS (Hou et al., 2019) in rice tissues were determined (detailed information is shown in **Supplementary material M1**).

Activities of enzymes involved in 2-OG biosynthesis, i.e., NADP-ICDH (Gálvez et al., 1994), isocitrate dehydrogenases (NAD-IDH) (Gálvez et al., 1994), and glutamate dehydrogenases (GDH) (Turano et al., 1996), were also measured (detailed information is shown in **Supplementary material M1**).

### 2.5 RNA extraction and RT-qPCR analysis

Real-time quantitative PCR (RT-qPCR) was used to quantify the expression levels of CNM-related enzymes in rice seedlings after SCN^−^ exposure. Total RNA was extracted from both the root and shoot of all rice samples by using an Ultrapure RNA Kit (CWBio, Taizhou, China). DNase I (CWBio, Taizhou, China) was used to remove genomic DNA contamination, if any, from RNA extract. Then, the total RNA was purified by an RNeasy MinElute Cleanup Kit (Qiagen, Hilden, Germany). Each sample was prepared in four independent biological replicates.

A total of 40 genes encoding enzymes or proteins activated in the CNM pathways were searched from the databases, including RGAP (http://rice.plantbiology.msu.edu/analyses_search_blast.shtml), NCBI (https://www.ncbi.nlm.nih.gov/), and RAPDB (http://rapdb.dna.affrc.go.jp/). Expression of genes was assayed after SCN^−^ exposure by RT-qPCR analysis, including PEPC (*Osppc1*, *Osppc2a*, *Osppc3*, and *Osppc4*), ERS (*OsERS1*, *OsERS2*, and *OsERS3*), TPS (*OsTPS1*, *OsTPS4*, *OsTPS5*, *OsTPS8*, and *OsTPS9*), SPS (*OsSPS1*, *OsSPS2*, *OsSPS4*, *OsSPS5*, and *OsSPS6*), NR (*OsNIA1*, *OsNIA2*, and *OsNR1*), NiR (*OsNiR1*, *OsNiR2*, and *OsNiR3*), GS (*OsGS1;1*, *OsGS1;2*, *OsGS1;3*, and *OsGS2*), NADP-ICDH (*OsICDH1*, *OsICDH2*, *OsICDH3*, and *OsICDH4*), NAD-IDH (*OsIDHc;2*, *OsIDHc;1*, *OsIDHa*, and *OsIDH1*), and GDH (*OsGDH1*, *OsGDH2*, *OsGDH3*, and *OsGDH4*). All genes primer sequences are listed in **Table S1**. RT-qPCR cycling conditions were as follows: 1) denaturation at 95°C for 10 s, 2) annealing at 58°C for 30 s, and 3) extension at 72°C for 32 s. This cycle was imitated 40 times. The RT-qPCR analysis was executed using the 7500 Fast Real-Time PCR system (Applied Biosystems, Foster City, CA, USA) and SYBR green chemistry. Rice GAPDH (glyceraldehyde-3-phosphate dehydrogenase, LOC_Os08g03290.1) was selected as the housekeeping gene (Yang et al., 2021). The standard 2^−ΔΔCT^ method was used to calculate the relative expression of each of the targeted genes (Schmittgen and Livak, 2008). All values were represented as cumulative means ± standard deviation of four independent replicates.

### 2.6 Identification of key regulatory genes in the CNM regulatory module

#### 2.6.1 Co-expression network analysis

In order to establish the CNM regulatory module with statistical significance, all CNM-related genes were uploaded to the STRING (https://version-10-5.string-db.org/) software, and the protein–protein interaction (PPI) networks (combined score >0.4) were constructed. Then, the modules (resolution = 0.8) with higher visualization were performed by the program Gephi 0.9.2 (Lin et al., 2022b).

#### 2.6.2 Estimation of the normcdf of CNM-related genes

In order to identify the key module activated in CNM in rice seedlings exposed to SCN^−^, the results from PCR tests were used to calculate the possibility of the occurrence of genes grouped in four different modules. We first converted the data through the functions of “COMPUTE x_new=SQRT(X)” or “COMPUTE x_new = LN(x)” in the SPSS software since they were non-normally distributed. Then, the normcdf was calculated statistically.

### 2.7 Data analysis

Tukey’s multiple range tests were used to assess the statistical significance at the level of 0.01 or 0.05. Different letters refer to the significant difference between the treatments and control (*p*< 0.05). The asterisk symbol refers to the significant difference between SCN^−^-treated and “SCN^−^ + 2-OG”-treated seedlings (*p*< 0.05).

## 3 Results

### 3.1 Relative growth rate of rice seedlings

**Figure 1:**
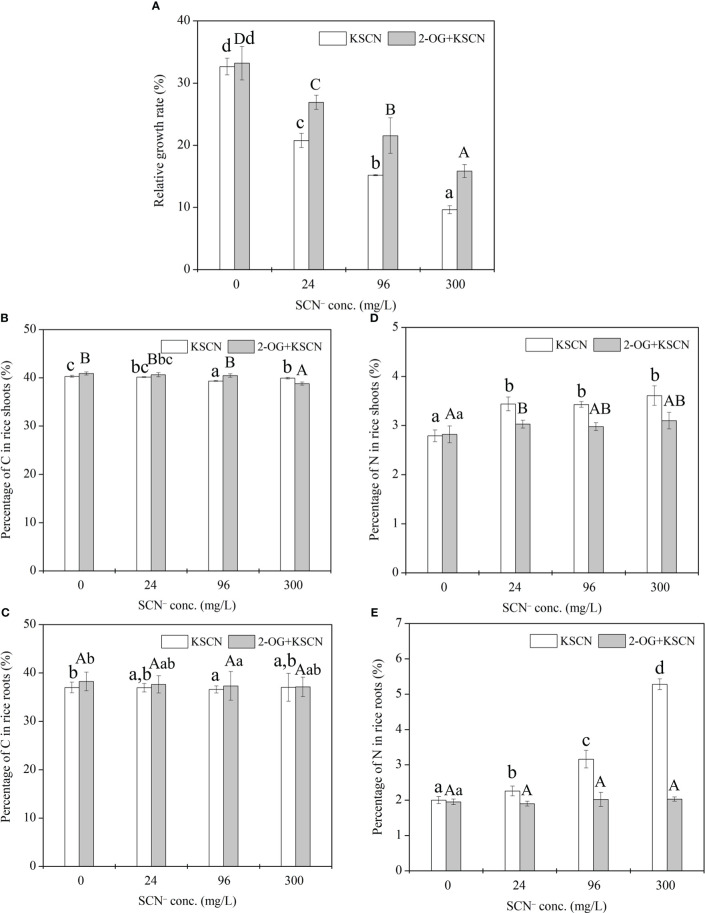
**(A)** Relative growth rate of rice seedlings under SCN^−^ exposure in the presence or absence of 2-OG. **(B)** The percentage of C in rice shoots. **(C)** The percentage of C in rice roots. **(D)** The percentage of N in rice shoots. **(E)** The percentage of N in rice roots. Values are the mean of four independent biological replicates ± standard deviation. Different letters refer to the significant difference between treatment and control (*p*< 0.05). 2-OG, 2-oxoglutarate.

A remarkable (*p*< 0.05) reduction in RGR of rice seedlings was observed at all SCN^−^ treatments after 3-day exposure in comparison to the control (**Figure 1A**). Similarly, in the case of “SCN^−^ + 2-OG” treatments in rice seedlings, a decrease in RGR that was visible in all treated plants reversed to that of control (*p*< 0.05). However, the RGR of rice seedlings under “SCN^−^ + 2-OG” treatments was significantly (*p*< 0.05) higher than that of SCN^−^ treatments, suggesting that the inoculation of 2-OG could mitigate the negative effect of SCN^−^ on plant biomass growth.

### 3.2 The total amount of C and N in rice seedlings

The C% in rice roots (shoots) was 36.99%, 36.94%, 36.58%, and 37.04% (40.31%, 40.15%, 39.35%, and 39.93%, respectively) under 0, 24, 96, and 300 mg SCN/L treatments, respectively. The application of exogenous 2-OG caused a negligible effect on the C% in rice tissues compared with their respective SCN^−^ treatments (**Figures 1B, C**). The N% in rice roots (shoots) was 2.0%, 2.26%, 3.16%, and 5.28% (2.79%, 3.44%, 3.43%, and 3.61%, respectively) under 0, 24, 96, and 300 mg SCN/L treatments, respectively, while application of exogenous 2-OG significantly decreased the N% in rice tissues compared with their respective SCN^−^ treatments (**Figures 1D, E**).

### 3.3 Response of CNM-related genes in rice plants

Mostly, more than one isogene was encoded with the specific enzyme in plants, in which the activity of the enzyme was regulated and/or governed by these isogenes together. However, each specific isogene does not carry the same weight during the regulation process, wherein there is always a master regulator gene (Yang et al., 2021), which chiefly controls the enzyme activity. Here, the upregulated master regulator genes in rice seedlings were described, while downregulated genes are not described in the following sections.

#### 3.3.1 Response of C metabolism-related genes

**Figure 2:**
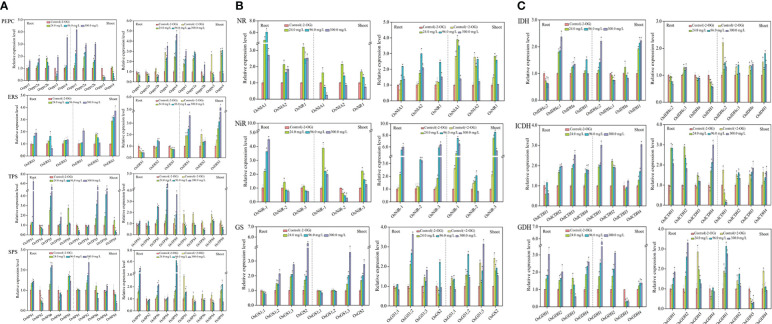
Response of CNM-related genes in rice roots and shoots under SCN^−^ stress in the presence or absence of 2-OG. **(A)** Response of C metabolism-related genes. **(B)** Response of N metabolism-related genes. **(C)** 2-OG synthesis-related genes. The asterisk symbol refers to the significant difference between SCN^−^-treated (24.0, 96.0, and 300.0 mg SCN/L) and SCN−+2-OG-treated (0, 24.0, 96.0, and 300.0 mg SCN/L) seedlings and control 1 (*p*< 0.05). CNM, carbon and nitrogen metabolism; 2-OG, 2-oxoglutarate.

As shown in **Figure 2A**, PEPC, upregulated genes in roots were *Osppc4*, *Osppc2a*, and *Osppc2b*, at all SCN^−^ treatments, while *Osppc1*, *Osppc2a*, and *Osppc2b* were upregulated in shoots. In roots of rice seedlings from the “SCN^−^ + 2-OG” treatments, *Osppc4* and *Osppc3* were upregulated, while *Osppc4*, *Osppc1*, and *Osppc2a* showed remarkable expression in shoots.

Expression of ERS-related genes is shown in **Figure 2A**, ERS, wherein *OsERS1*, *OsERS2*, and *OsERS3* were generally upregulated in SCN^−^-exposed rice parts, i.e., in roots and shoots. However, the expression levels of *OsERS1*, *OsERS2*, and *OsERS3* showed raised pattern in shoots of rice plants from the “SCN^−^ + 2-OG” treatments.

**Figure 2A**, TPS, depicts that higher expression levels of TPS-related genes of *OSTPS5* and *OSTPS1* were prominent in roots in SCN^−^ treatments, while *OSTPS5* and *OSTPS8* had greater expression in shoots. Interestingly, when rice seedlings were pretreated with 2-OG, *OSTPS5*, *OSTPS8*, and *OSTPS9* were upregulated in roots. However, the expression levels of five TPS isogenes in shoots conferred a variance tendency, which reinforced at 0 mg SCN/L and then declined at 24 mg SCN/L.

Upregulation of three SPS isogenes (*OsSPS1*, *OsSPS3*, and *OsSPS5*) was observed in roots in all SCN^−^ treatments (**Figure 2A**, SPS), while only *OsSPS2* was upregulated in shoots. Differential expression patterns were found in SPS-related genes of roots in “SCN^−^ + 2-OG” treatments. The expression levels of *OsSPS1*, *OsSPS3*, and *OsSPS5* in roots from the “SCN^−^ + 2-OG” treatments were higher than those of SCN^−^ treatments. *OsSPS1* and *OsSPS5* were upregulated in shoots of “SCN^−^ + 2-OG” treatments, which differed from SCN^−^ treatments.

#### 3.3.2 Response of N metabolism relative genes

Positive expressions of NR genes, i.e., *OsNIA1*, *OsNIA2*, and *OsNR1*, were observed in roots after SCN^−^ exposure (**Figure 2B**, NR). However, the expression levels of *OsNIA1*, *OsNIA2*, and *OsNR1* in shoots showed a disparate trend with an initial escalation from 24 mg SCN/L and then dropped at 96 mg SCN/L. In the case of rice seedlings from the “SCN^−^ + 2-OG” treatments, higher expression levels of *OsNIA1*, *OsNIA2*, and *OsNR1* were observed in rice shoots.

Within NiR genes, only *OsNiR-1* was upregulated in roots at all SCN^−^ treatments (**Figure 2B**, NiR), while upregulation of *OsNiR-1* and *OsNiR-3* was detected in shoots. Interestingly, the expression levels of *OsNiR-1*, *OsNiR-2*, and *OsNiR-3* conferred an accelerating pattern in both roots and shoots of the “SCN^−^ + 2-OG” treatments.

Differential expression of GS-related genes was observed between roots and shoots, with significant upregulation of *OsGS2*, *OsGS1;2*, and *OsGS1;3* in roots and shoots (*OsGS2* and *OsGS1;3*) (**Figure 2B**, GS). However, the expression levels of *OsGS2*, *OsGS1;2*, *OsGS1;1*, and *OsGS1;3* showed linear inclination with increasing SCN^−^ concentrations in both rice tissues by inoculation of 2-OG.

#### 3.3.3 Genes involved in the biosynthesis of 2-OG

Transcriptional changes of NAD-IDH genes are shown in **Figure 2C**, IDH. As apparent from the figures, *OsIDHa* and *OsIDHc;1* were upregulated in roots, while *OsIDHc;2* and *OsIDH1* were overexpressed in shoots. When rice seedlings were pretreated with 2-OG, upregulation of *OsIDHc;1* was observed in roots, and *OsIDHa*, *OsIDHc;2*, and *OsIDH1* were upregulated in shoots.

As presented in **Figure 2C**, ICDH, *OsICDH2*, *OsICDH3*, and *OSICDH4* were significantly (*p*< 0.05) upregulated in roots after SCN^−^ exposure, while positive expressions (*p*< 0.05) of *OsICDH1*, *OsICDH2*, and *OsICDH4* were observed in shoots. Interestingly, *OsICDH1*, *OsICDH2*, and *OsICDH4* in roots presented an upregulated pattern in the “SCN^−^ + 2-OG” treatments and the expression levels of *OsICDH2*, *OsICDH3*, and *OsICDH4* were remarkable in shoots.

Upregulation of *OsGDH1*, *OsGDH2*, and *OsGDH4* was observed in both rice tissues after SCN^−^ exposure (**Figure 2C**, GDH), while significantly (*p*< 0.05) higher expressions of *OsGDH1*, *OsGDH2*, and *OsGDH3* were observed in roots, and significant upregulation of *OsGDH1*, *OsGDH2*, and *OsGDH4* was detected in shoots of the “SCN^−^ + 2-OG” treatments.

### 3.4 Response of CNM-related enzyme activities

#### 3.4.1 Response of C metabolism-related enzyme activities

**Figure 3:**
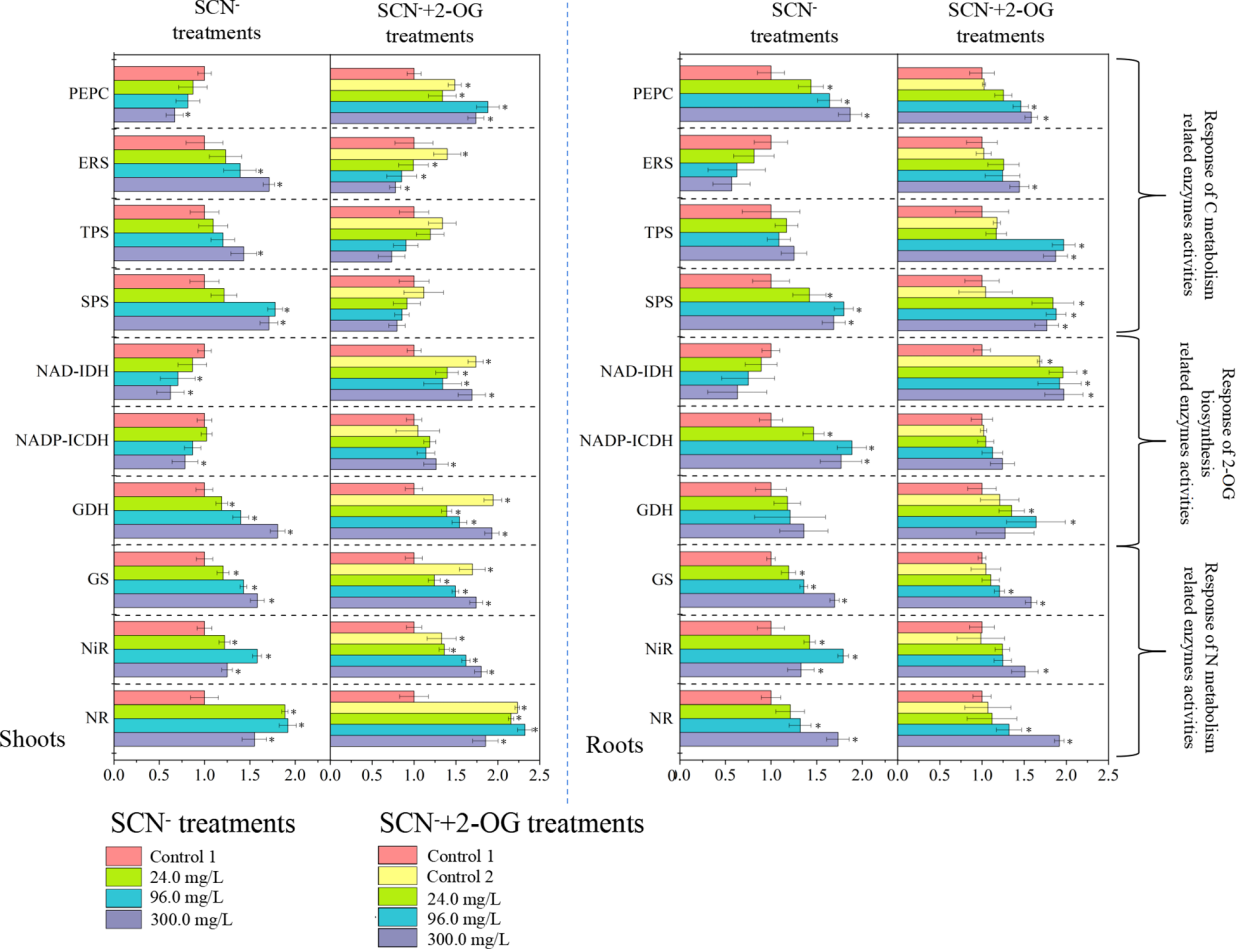
Response of CNM-related enzyme activities in rice roots and shoots under SCN^−^ stress in the presence or absence of 2-OG. The asterisk symbol refers to the significant difference between SCN^−^-treated (24.0, 96.0, and 300.0 mg SCN/L) and SCN−+2-OG-treated (0, 24.0, 96.0, and 300.0 mg SCN/L) seedlings and control 1 (*p*< 0.05). CNM, carbon and nitrogen metabolism; 2-OG, 2-oxoglutarate.

The activities of CNM-related enzymes were assayed in SCN^−^ and “SCN^−^ + 2-OG” treatment plants (**Figure 3**). The activity of PEPC in roots was affirmatively increased (*p*< 0.05) after SCN^−^ exposure compared with the control, while the activity of PEPC in shoots presented a downward tendency. The activity of ERS in roots was significantly inhibited (*p*< 0.05) after SCN^−^ exposure in comparison with the control, while the activity of ERS was increased in shoots. Activities of TSP and SPS presented significant increasing patterns in both roots and shoots in the presence of SCN^−^ stress (*p*< 0.05). Under “SCN^−^ + 2-OG” treatments, activities of PEPC, ERS, TSP, and SPS intensified in roots compared to control (*p* > 0.05), while activities of ERS, TSP, and SPS showed a decrement in shoots unlike control (*p* > 0.05).

#### 3.4.2 Response of N metabolism-related enzyme activities

Activities of NR and NiR in roots presented an exponential emulate compared with the control (*p*< 0.05), while activities of NR and NiR in shoots illustrated an inverted “U” shape curve under SCN^−^ stress (**Figure 3**). The analogous activity of GS was noticed in both roots and shoots of SCN^−^ stress (*p*< 0.05), following linearity with stress concentration. Under “SCN^−^ + 2-OG” treatments, activities of NR, NiR, and GS showed a similar pattern of increment in both roots and shoots (*p*< 0.05).

Overall, the activities of all selected enzymes in “SCN^−^ + 2-OG” treatments were generally higher than those of SCN^−^ treatments. Exogenous 2-OG had a pronounced impact on enzyme activities in shoots, unlike its counterpart. To reveal the regulation mechanism of exogenous 2-OG on the CNM in rice plants under SCN^−^ exposure, we distinguished the effects of exogenous 2-OG on C and N metabolism in rice tissues.

#### 3.4.3 Response of 2-OG biosynthesis-related enzyme activities

The activity of NAD-IDH was inhibited significantly (*p*< 0.05) in both roots and shoots under SCN^−^ exposure compared with the control (**Figure 3**). The activity of NADP-ICDH in SCN^−^-exposed roots was prominently enhanced; nevertheless, the scenario was reversed in the case of shoots (*p*< 0.05). Elevated activity of GDH was observed in both roots and shoots in the presence of SCN^−^ (*p*< 0.05). Under “SCN^−^ + 2-OG” treatments, the activities of NAD-IDH, NADP-ICDH, and GDH were generally increased in rice tissues, except for GDH in shoots of rice seedlings. These results indicated that the modification mechanism of exogenous 2-OG on CNM-related enzymes in rice seedlings under SCN^−^ exposure varied greatly.

### 3.5 Identification of key regulatory genes in the CNM regulatory module

#### 3.5.1 Co-expression analysis of CNM-related genes

**Figure 4:**
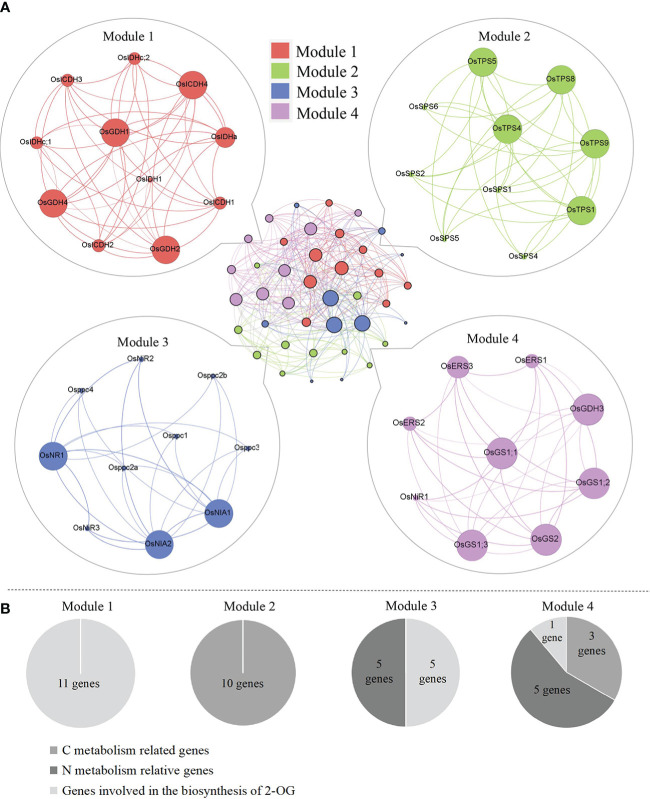
**(A)** Genetic matrix integrative analysis of CNM-related genes. **(B)** The number of CNM genes in each matrix. CNM, carbon, and nitrogen metabolism.

Plants have evolved the coordinated actions responsible for their diverse physiological processes *via* either direct or indirect gene connections. In order to elucidate the functional module of genes activated in the CNM process, a co-expression network was performed by the STRING program, and four modules were obtained. Detailed information on gene interaction strengths in these four modules is given in **Table S2**. Interestingly, all modules had similar interaction contributions, namely Module 1 (25.0%), Module 2 (25.0%), Module 3 (27.5%), and Module 4 (22.5%) (**Figure 4A**). We also noticed that the genes grouped in Modules 1 and 2 were involved in C metabolism (11 genes) and biosynthesis of 2-OG (10 genes), respectively; genes categorized in Module 3 were responsible for N metabolism (five genes) and biosynthesis of 2-OG (five genes). In addition, genes grouped in Module 4 were activated in C metabolism (one gene) and N metabolism (five genes) and biosynthesis of 2-OG (three genes) (**Figure 4B**).

#### 3.5.2 The occurrence probability of CNM-related genes

**Figure 5:**
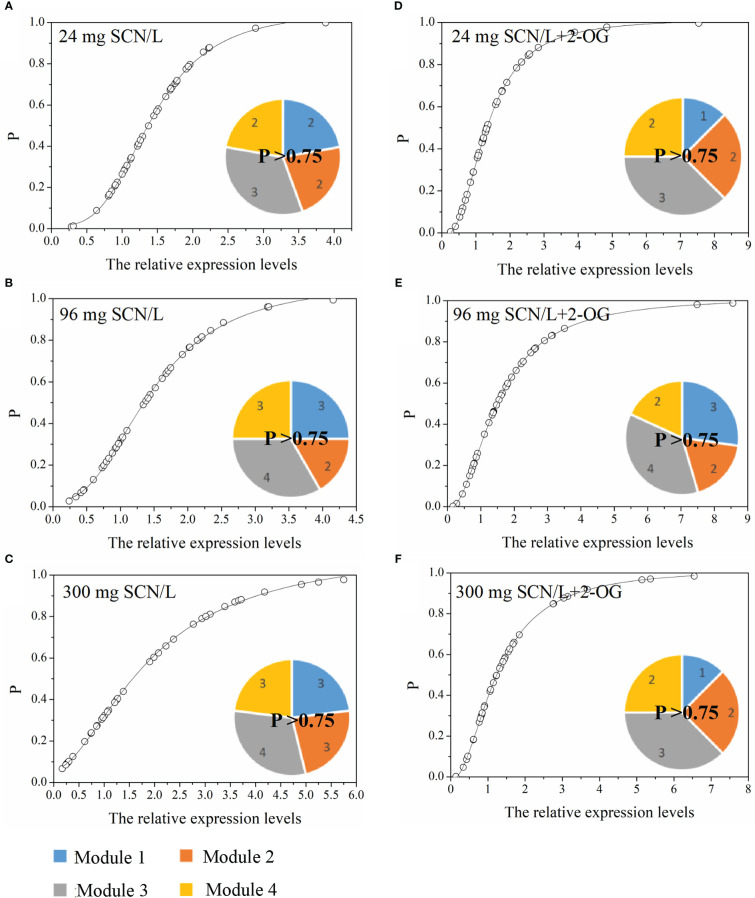
Occurrence P (*p* > 0.75) of CNM-related genetic expression in rice shoots under **(A)** 24 mg SCN/L, **(B)** 96 mg SCN/L, and **(C)** 300 mg SCN/L treatments and under **(D)** 24 mg SCN/L + 2-OG, **(E)** 96 mg SCN/L + 2-OG, and **(F)** 300 mg SCN/L + 2-OG treatments. CNM, carbon and nitrogen metabolism; 2-OG, 2-oxoglutarate.

The normcdf of rice shoots was quite different between under SCN^−^ and SCN−+2-OG treatments, based on the non-linear regression (**Figure 5**). Herein, the threshold for the highest occurrence probability was set, *p* > 0.75. Therefore, Module 3 showed the highest occurrence probability, suggesting that the exogenous application of 2-OG mainly regulated the expression of genes activated in the N metabolism and 2-OG synthesis to modify the imbalance of CNM in rice plants imposed by SCN^−^ exposure. In fact, a similar conclusion was also reached in the analysis of C and N fractions in rice tissues. We noticed that the change of C fraction in rice shoots was almost constant (**Figures 1B, C**), while the change of N in rice shoots was evident between SCN^−^ treatments and “SCN^−^ + 2-OG” treatments (**Figures 1D, E**).

## 4 Discussion

### 4.1 Exogenous 2-OG promotes plant growth *via* regulating CNM during SCN^−^ exposure

The growth and development of plants are tightly coordinated with the balance of cellular CNM (Zheng, 2009). Once plants suffer from environmental stresses, the CNM in plants could be disrupted, thereby causing an imbalance of CNM and eventually resulting in a reduction in plant growth (Reddy et al., 2004). In this current study, the imbalance of CNM in rice seedlings due to SCN^−^ exposure was judged by the relative growth rate (**Figure 1A**), in which SCN^−^ exposure led to a significant reduction in RGR of rice seedlings (*p*< 0.05), indicating a severe impact on the balance of CNM in rice seedlings under SCN^−^ stress. Also, we found that SCN^−^ exposure significantly affected the percentage of N in rice seedlings (**Figures 1D, E**). This is because SCN^−^ exposure can result in the dysfunction of chloroplast (Yang et al., 2021) and repress the activities of NR, GS, and glutamate synthase (GOGAT) in rice seedlings (Lin et al., 2022a). In addition, SCN^−^-treated rice seedlings with 2-OG supplied had significantly (*p*< 0.05) higher RGR than those without exogenous 2-OG, suggesting a positive effect of exogenous 2-OG on the RGR of rice seedlings corresponding to SCN^−^ exposure. It is known that 2-OG is a decisive chemical involved in the homeostasis of CNM in higher plants (Yuan et al., 2007). Also, exogenous 2-OG enhances photosynthesis and increases the levels of C-skeletons in rice plants, thus affecting the N metabolism (Yuan et al., 2007; Zheng, 2009). Therefore, the imbalance of CNM in rice seedlings due to SCN^−^ exposure could be positively modified by exogenous 2-OG.

### 4.2 Modification of 2-OG in balancing CNM in SCN^−^-treated rice plants

Herein, the main N source present in the nutrient solution to support plant growth is NO_3_^−^. In plants, only a small fraction of NO_3_^−^ is assimilated in roots after uptake, and a greater part is translocated into shoots and assimilated into NH_4_^+^ and amino acids. Photosynthesis in the chloroplast is a major process for C metabolism in plants during their entire period of life (Lemaitre et al., 2007). Therefore, modification of 2-OG on CNM-related genes and enzyme activities in rice shoots will be discussed accordingly.

#### 4.2.1 Effects of SCN^−^ on innate 2-OG synthesis

It is known that there are three innate routines for the biosynthesis of 2-OG in plants, in which the PEPC pathway and the NADP-ICDH/NAD-IDH pathway belong to C metabolism, and the GDH pathway is mainly involved in N metabolism. In this study, we focused on correlating and perceiving the most competent pathway in controlling the generation of 2-OG and regulating the imbalance of CNM in rice seedlings caused by SCN^−^ exposure.

The PEPC pathway in plants is an anaplerotic reaction to replenish the tricarboxylic acid (TCA) cycle with intermediates that are withdrawn for different biosynthesis pathways and N metabolism (Lemaitre et al., 2007). For example, PEPC is able to catalyze phosphoenolpyruvic acid (PEP) into 2-OG, and 2-OG synthesis from malate can be suppressed by the knockdown of *Osppc4*, therefore causing a decrease in plant growth and leaf area (Masumoto et al., 2010), suggesting that *Osppc4* is crucial for the growth of rice plants. In the present study, significant downregulation of *Osppc4* was observed in shoots of rice seedlings after SCN^−^ exposure (**Figure 2C**). The activity of PEPC was decreased in shoots after SCN^−^ treatments (**Figure 3**), suggesting that the synthesis of 2-OG in shoots from the PEPC pathway was repressed by SCN^−^ exposure.

The second pathway of 2-OG generation is the NADP-ICDH/NAD-IDH pathway (Ferrario-Mery et al., 2001), in which citrate can be either exported from mitochondria to cytosol for 2-OG synthesis by cytosolic enzymes aconitase NADP-ICDH or transformed into 2-OG in mitochondria by TCA cycle enzyme aconitase NAD-IDH (Yuan et al., 2007). Indeed, NAD-IDH is often regarded as a major governing point in plants (Lemaitre et al., 2007), which is encoded with one single gene *OsIDHa* in rice plants (Lancien et al., 1998). Herein, significant downregulation of *OsIDHa* was detected in shoots after SCN^−^ exposure (**Figure 2C**), suggesting that SCN^−^ exposure could inhibit the expression of *OsIDHa* in shoots. A significant correlation was obtained in the enzymatic assay of NAD-IDH, wherein a decrease in the activity of NAD-IDH was observed in shoots of rice seedlings exposed to SCN^−^ (**Figure 3**). Results from both C-related pathways indicated that SCN^−^ exposure significantly repressed both pathways to produce 2-OG, thereby causing a severe impact on the C metabolism and breaking the balance of CNM.

The third source to produce 2-OG is from the GDH pathway, in which the oxidative deamination of glutamate (Glu) into 2-OG is catalyzed by GDH. It has been reported that exogenous 2-OG increased the activities of GDH in wheat seedlings and promoted yield productivity under drought stress (Lancien et al., 1998). Additionally, GDH plays a unique role in the formation of NH_4_^+^ and 2-OG during the assimilation of Glu (Lodwig et al., 2003). We found that the expression levels of *OsGDH1* and *OsGDH2* were significantly upregulated in shoots after SCN^−^ treatments (**Figure 2C**). The change of GDH activity in shoots was constructive against SCN^−^ exposure (**Figure 3**). These results indicated that SCN^−^ exposure does not disturb the conversion of Glu into 2-OG through the activation of GDH. Combined with the results from C-related pathways of 2-OG, we have sufficient reasons to conclude that the imbalance of CNM in rice seedlings was evident due to SCN^−^ exposure through repressing the two C-related pathways.

#### 4.2.2 Effects of SCN^−^ on innate 2-OG synthesis in the presence of exogenous 2-OG

Compared with SCN^−^ treatments (**Figure 2C**), the expression levels of *Osppc4* in shoots of rice seedlings under “SCN^−^ + 2-OG” treatments were significantly upregulated, and the activity of PEPC in shoots was also positively responsive, suggesting that the application of 2-OG enhances the enzyme activity of PEPC and might stimulate the generation of 2-OG. A similar conclusion was also predicted in the second pathway of 2-OG generation due to the application of exogenous 2-OG, wherein a correlation between upregulated expression of *OsIDHa* and increases in NAD-IDH activity was obtained. Additionally, the expression levels of *OsGDH1* and *OsGDH2* in shoots of rice seedlings fed with 2-OG were significantly upregulated, and an increase of GDH activity in shoots was also detected (**Figure 3**), suggesting that the conversion of Glu into 2-OG was independent of the application of 2-OG. Co-expression network analysis showed that the GDH-related genes in Module 1 had a higher connection degree with others (**Figure 4A**). Apparently, these results indicated that the two C-related pathways were significantly activated due to the application of 2-OG, in which sufficient 2-OG in plant cells was able to modify the imbalance of CNM in rice seedlings caused by SCN^−^ exposure, subsequently decreasing the negative impact on rice seedlings, which was judged by a measurable increase in biomass growth of rice seedlings from the SCN^−^-treated rice seedlings with 2-OG, compared with the SCN^−^-treated rice seedlings without 2-OG.

### 4.3 Responses of other CNM-related enzymes and genes in rice plants after SCN^−^ exposure

#### 4.3.1 Effects of SCN^−^ on C metabolism in rice plants

The TPS and SPS are primary targeted cytosolic enzymes involved in the C metabolism (Coruzzi and Zhou, 2001). Previous studies indicated that the expressions of *OsTPS2*, *OsTPS5*, and *OsTPS6* in rice were negatively correlated with sucrose starvation (Wang et al., 2007). The expression of *SPS* genes was positively correlated with non-structure carbohydrate content in the leaf, wherein *OsSPS1* expression and SPS activity were affirmatively corresponding to spike number and grain yield (Li and Cui, 2018). However, another study showed that mRNA levels of *OsSPS1* and *OsSPS6* were negatively correlated with sucrose concentrations (Yonekura et al., 2013). In the present study, upregulation of *OsTPS5* was detected in both rice tissues under SCN^−^ stress (**Figure 2**). In addition, upregulated *OsSPS1* was also evident in both roots and shoots. Meanwhile, increases in TPS and SPS activities in rice tissues were detectable (**Figure 3**). These results indicated that SCN^−^ exposure stimulated the expression of C metabolism-related master regulation genes, thus regulating the enzyme activities. Enzyme ERS has an important role in maintaining the physiological homeostasis of amino acids and C metabolism as well as redox status (Yang et al., 2018). Like TPS and SPS, upregulation of *OsERS1* was distinguished in both rice tissues after SCN^−^ stress (**Figure 2**). Also, we observed that responses of *OsERS1* to SCN^−^ exposure were identical to the enzyme activity of ERS in rice tissues (**Figure 2**).

#### 4.3.2 Effects of SCN^−^ on N metabolism in rice plants

NR, NiR, and GS are three key enzymes involved in N assimilation. The conversion of NO_3_^−^ into NH_4_^+^ is catalyzed by the enzymes NR and NiR, which is a rate-limiting step in NO_3_^−^ assimilation. In the present study, three isogenes of NR (*OsNIA1*, *OsNIA12*, and *OsNR1*) and NiR (*OsNiR-1*, *OsNiR-2*, and *OsNiR-3*) showed a declining expression pattern in shoots after SCN^−^ exposure, of which SCN^−^ treatments at 24.0 and 96.0 mg/L positively regulated transcriptional changes in *OsNR1*, and 300.0 mg SCN/L treatment demonstrated a negative response (**Figure 2**). Enzymatic assay showed that activities of NR and NiR had a positive correlation with gene expression (**Figure 3**), suggesting that low-to-moderate concentrations of SCN^−^ exposure might stimulate the conversion of NO_3_^−^, and higher SCN^−^ concentrations had a negative effect on this process, whereas a similar conclusion was also reached by Lin et al. (2022a). Another crucial enzyme in N metabolism is GS, which is responsible for converting NH_4_^+^ into glutamine. In this study, almost all GS genes were upregulated in the SCN^−^ treatments (**Figure 2**). It is established that GS in most plants occurs as GS2 in plastids and GS1 in the cytosol. The role of GS1 is to assimilate NH_4_^+^ in roots and reassimilate NH_4_^+^ in leaves, whereas GS2 is mainly responsible for assimilating NH_4_^+^ derived from NO_3_^−^ reduction in plastids (Lin et al., 2022a). The enzymatic assay also showed that activities of GS had a positive correlation to gene expression (**Figure 3**), suggesting that SCN^−^ treatments do not inhibit the activity of GS and subsequently increase the conversion of NH_4_^+^.

#### 4.3.3 Effects of exogenous 2-OG on CNM in rice plants under SCN^−^ stress

Compared with SCN^−^ treatments (**Figure 2**), exogenous 2-OG decreased the expression of *OsTPS5* in shoots of rice seedlings after SCN^−^ exposure. Similarly, the downregulation of *OsSPS6* and *OsERS1* was also detectable. Enzymatic assays indicated that the application of 2-OG decreased the activities of TPS, SPS, and ERS in shoots of rice seedlings (**Figure 3**). A co-expression network analysis showed that the C metabolism-related genes in Module 2 and Module 4 had a lower connection degree with others (**Figure 4A**). However, we noticed that NR and NiR genes were upregulated in SCN^−^-treated rice seedlings inoculated with 2-OG, wherein enzymes of NR and NiR were positively responsive to 2-OG application, indicating that the application of 2-OG had a positive impact on the conversion of NO_3_^−^. A previous study also reported that feeding of 2-OG increased transcripts of the NR gene in tobacco leaf (Ferrario-Mery et al., 2001). Additionally, under “SCN^−^ + 2-OG” treatments, the expression levels of *OsGS1;2* were significantly higher than those under SCN^−^ treatments, suggesting that the application of 2-OG also increases the conversion of NH_4_^+^ derived from the main N source of NO_3_^−^ supplied. A co-expression network analysis showed that the N metabolism-related genes in Module 3 and Module 4 had a higher connection degree with others (**Figure 4A**). These results indicated that sufficient 2-OG in plant cells can modify the imbalance of N metabolism-related genes in rice seedlings caused by SCN^−^ exposure.

## 5 Conclusion

**Figure 6:**
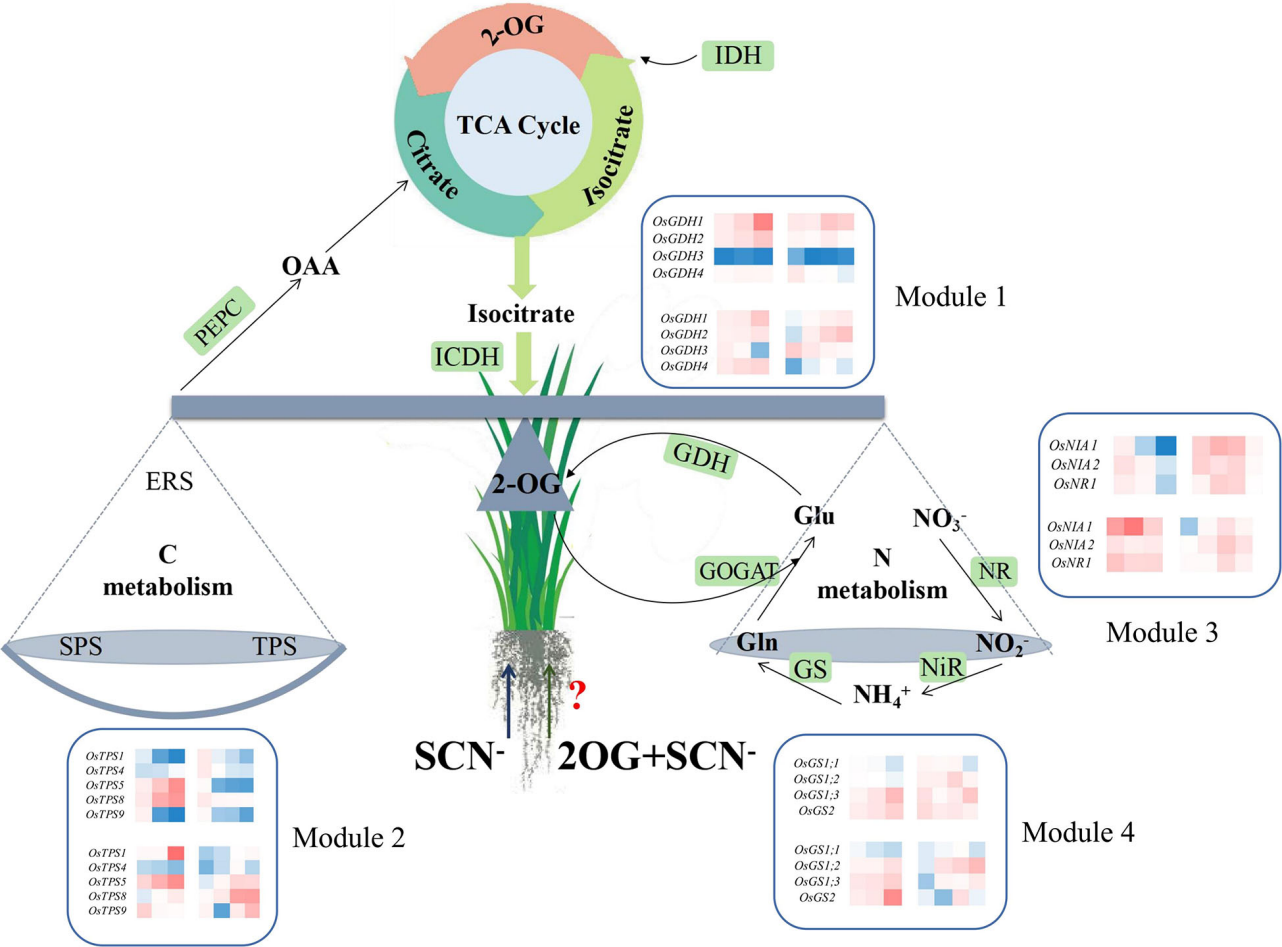
Overview of exogenous 2-OG-mediated CNM in rice tissues under SCN^−^ stress. 2-OG, 2-oxoglutarate; CNM, carbon and nitrogen metabolism.

The balance of CNM in rice seedlings can be broken by SCN^−^ exposure, resulting in a significant reduction in the biomass growth of rice seedlings. The application of exogenous 2-OG showed a positive regulatory effect on the imbalance of CNM in rice seedlings under SCN^−^ stress. Higher connection degrees of genes in each module in rice plants under SCN^−^ and “SCN^−^ + 2-OG” treatments are marked in **Figure 6**. Although our findings provide new insight into the role of exogenous 2-OG in minimizing the negative effect of SCN^−^ exposure on rice plants through regulating the pathways involved in CNM, the global molecular map of regulatory genes involved in CNM remains unclear. Further comprehensive studies are needed to experimentally prove the influence of exogenous 2-OG as a chemical regulator on the quality and quantity of agricultural crops through the “omics” technology, such as transcriptome, proteome, and metabolome.

## Data availability statement

The original contributions presented in the study are included in the article/**Supplementary Materials**. Further inquiries can be directed to the corresponding author/s.

## Author contributions

X-ZY: conceptualization, methodology, supervision, writing-reviewing and editing, and funding acquisition. Y-XF: writing-original draft preparation and visualization. LY: investigation. Y-JL: investigation, data analysis, visualization, and software. YS: data analysis, visualization, and software. All authors contributed to the article and approved the submitted version.
