# Supplementary material for: Merging the occurrence possibility into gene co-expression network deciphers the importance of exogenous 2-oxoglutarate in improving the growth of rice seedlings under thiocyanate stress
Source: Front Plant Sci. 2023 Feb 23;14:1086098. doi: 10.3389/fpls.2023.1086098 (PMC9995760; doi:10.3389/fpls.2023.1086098)
Supplement: Supplementary file 2 [file Table_1.doc]

***Supporting information on***

**Merging the occurrence possibility into gene co-expression network deciphers the importance of exogenous 2-oxoglutarate in improving the growth of rice seedlings under thiocyanate stress**

Yu-Xi Feng^1^, Li Yang^1^, Yu-Juan Lin, Ying Song, Xiao-Zhang Yu^*^

College of Environmental Science & Engineering, Guilin University of Technology, Guilin 541004, People’s Republic of China

**^*^Corresponding author**

Prof. Dr. Xiao-Zhang Yu. ORCID ID: 0000-0001-7846-5017

Phone: +86 7735897016. E-mail: [xzyu@glut.edu.cn](mailto:xzyu@glut.edu.cn)

^1^ The authors contribute equally to this work.

**Supporting information M1**

1. **NR and NiR assay**

Plant tissue was cut into small pieces and ground with sand and grinding medium (4 mL g^-1^ FW) in a chilled mortar and pestle. The grinding medium consisted of 0.1 M Tris-HCl buffer (pH7.5), 10 mM cysteine, 1 mM EDTA, and 5 μM flavin adenine dinucleotide. The homogenate was centrifuged at 15000 g for 25 min and the resulting supernatant was used for determination of enzyme activities. The whole extraction procedure was carried out at 4°C.

Nitrate reductase activity was estimated by measuring NO_2_^-^ formed in an assay system con-taining 0.1 M phosphate buffer (pH 7.5), 1 mM EDTA, 10 mM KNO_3_, the enzyme preparation, and 0.3 mM NADH, in a final volume of 1 mL. After 20 min incubation at 30°C, the reaction was terminated by addition of 0.1 mL of 1 M barium acetate and 1.9 mL of 96% (v/v) ethanol. After vigorous shaking in a mixer, the mixture was left to stand for 5 min at 4°C and centrifuged at 2300 g for 5 min, and then NO_2_ measured on an aliquot from the clear supernatant solution by addition of 1 mL of sulphanilamide reagent followed by 1 mL of N-(1-naphthyl) ethylenediamine reagent. Nitrite reductase activity was measured as the disappearance of NO_2_^-^ in the assay mixture by incubating an aliquot of the extract for 30 min at 30°C with 75mM Tris-HCl buffer (pH 7.5), 2mM NaNO2, and 0.75 mM methyl viologen reduced by 0.3 mL Na_2_S_2_O_4_ (8mg mL^-1^ in 0.3 M NaHCO_3_) , in a total volume of 2 mL (Ahanger et al., 2021; Lin et al., 2022)

1. **GS assay**

The enzyme extraction buffer for GS contained 0.025 mol L-1 tris-HCl (pH 7.2), 0.5 mmol L-1 EDTA, and 50 mmol L-1 K_2_SO_4_. The homogenates were centrifuged at 20 000 × g for 15 min and the supernatants were used for the enzyme assays; 1.2 mL of the aliquots were added to a 1.6 mL preincubation mixture containing 100 mmol L^-1^ imidazole (pH 7.0), 37.5 mmol L-1 ATP-Na, 75 mmol L-1 glutamate, and 50 mmol L-1 MgSO_4_. After 5 min of pre-incubation, the reaction was started by the addition of 70 mmol L-1 NH_2_OHHCl. Fifteen minutes later, the reaction was terminated with acidic FeCl_3_ (20 g L^-1^ TCA and 35 g L^-1^ FeCl_3_ in 20 g L^-1^ HCl). The activity of GS was measured in vitro as a synthetase reaction by the formation of γ-glutamyl hydroxamate. One unit is defined as the amount required to catalyze the synthesis of 1 mmol of γ-glutamyl hydroxamate per minute (Huo et al., 2019).

1. **SPS assay**

Sucrose phosphate phosphatase (SPS) was extracted from rice roots and shoots. All fresh samples were frozen in liquid nitrogen with buffer solution, and the supernatants were collected using centrifugation at 3000g for 20 min. More specifically, to analyze the activities of SPS and SS, frozen fresh tissues were ground in liquid nitrogen with 3 mL of HEPES-NaOH buffer (pH 7.5) containing 15 mM MgCl_2_, 25 mM fructose 6-phosphate, 25 mM glucose 6-phosphate, and 25 mM UDPglucose. To determine the activities of SSS, frozen fresh tissues were ground in liquid nitrogen with 3 mL of extraction buffer containing 10 mM Tricine-NaOH buffer (pH 8.0), 8 mM MgCl2, 2 mM EDTA, 50 mM mercaptoethanol, 12.5% glycerinum, and 5% PVP-K40. The extract was used for quantifying SPS, SS, and SSS activities using the ELISA Kit purchased from Nanjing Jiancheng Biology Engineering Institute (Feng et al., 2019).

1. **TPS assay**

TPP was assayed at 37°C by measuring the production of [^14^C]trehalose from [^14^C]trehalose-6-phosphate. Crude extracts were prepared in 25 mM Tris-HC1, pH 7.4, containing 5.5 mM MgCl_2_. Samples were diluted to a protein concentration of 1 mg/mL in extraction buffer containing 1 mg/mL BSA. Standard assay mixtures (50 μL final volume) contained 27.5 mM Tris-HCl, pH 7.4, 5.5 mM MgCl_2_, 1 mg/mL BSA, and 0.55 mM trehalose-6-phosphate (specific activity 854 cpm/nmol). Reactions were initiated by the addition of 5 juL of enzyme and terminated after 1 h by heating for 5 min in boiling water. AG1-X8 (formate) anion-exchange resin (Bio-Rad) was added and the reaction mixtures were centrifuged after 20 min of equilibration at room temperature. The radioactivity in the supernatant of the samples (400 luL) was measured by liquid scintillation counting. (Goddijn et al., 1997)

1. **PEPC****, GDH, ICDH, and IDH assay**

Enzymes were extracted from plant material that had been harvested at the time points indicated and immediately frozen in liquid nitrogen. The frozen leaf material was then reduced to a fine powder in a mortar withliquid nitrogen and stored at -80℃ until assay. All crude enzyme extractions were performed at 4 ℃.

PEPC was extracted in 0.1 M Tris-HCl (pH 8.0) 20% glycerol, 10 mM MgCl2, 5 mM NaF, 1 mM phenylmethylsulfonyl fluoride (PMSF), 2 nM okadaic acid, 20 lM leupeptin, 16 μM chymostatin and 2% (w/v) polyvinylpyrrolidone. The homogenate was centrifuged at 12,000 g for 5 min and the supernatant was used as the crude extract. PEPC activity was measured spectrophotometrically at two different pH values (pH 8.0, and 7.1) at 30 ℃ in a total reaction volume of 1 ml containing 50 mM Hepes-KOH, 5 mM MgCl_2_, 5 mM NaF, 1 mM NaHCO_3_, 0.2 mM NADH, 3 mM PEP, 10 U ml^–1^ malate dehydrogenase (Boehringer Mannheim, Germany). The apparent PEPc phosphorylation state was measured as the ratio of the activity measured under optimal conditions (pH 8.0) to that measured under sub-optimal conditions (pH 7.1), as described by Osuna et al. (1996).

The IDH activity of mitochondrial-enriched protein extracts was measured with a spectrophotometer by following the absorption change at 340 nm due to the production of NADH during the reaction. This was carried out in a buffer containing 50 mM HEPES, pH 7.4, 5 mM MnCl2, 10 mM isocitrate, 10 mM NADH, and 20% glycerol. ICDH was extracted in 0.1 M potassium phosphate buffer (pH 7.5), 1 μM leupeptin, 2 mM 2-mercaptoethanol, 1% (w/v) polyvinylpyrrolidone. Total ICDH activity was measured spectrophotometrically by following the reduction of NADP at 340 nm at 30℃ in 0.1 M potassium phosphate buffer (pH 7.5), 5 mM MgCl2, 2.5 mM isocitrate and 0.25 mM NADP as described by Galvez et al. (1994). NADH and NAD-dependent GDH reactions were measured as described by Turano et al. (1996).

1. **ERS assay**

The enzymic activities were measured by the rate of aminoacyl-tRNA formation under standard conditions. The incubation mixture (100 μl) contained 55 mM Hepes buffer at pH 7.8, 0.044 mM bovine serum albumin, 1.2 mM glutathione, 2mM ATP, 10mM KCl, 8μM L-[^14^C]glutamic acid (250 Ci/mol), 15 mM MgCl_2_, for chloroplast enzyme and 10 mM for germ enzymes, 1.6 μM E. coli tRNA^Glu^ or 0.8 μM rice tRNAG^Glu^ according to the nature of the enzymes and variable amounts of enzymic proteins in order to observe initial velocities. The incubation was carried out at 25 ℃ for 3 ~ 30 min. The assays were initiated by addition of the enzyme to the incubation mixture. The radioactive glutamyl-tRNAs formed were treated according to the method of Mans and Novelli. The radioactivity was counted in a Beckman LS 7000 scintillation counter with 60 %,efficiency for I4C. The unit of enzymic activity (U) is defined as the amount catalyzing the formation of 1 nmol L-[^14^C]glutamyl-tRNA/ min. (Ratinaud et al., 1983)

**Table S1** Sequence of forward and reverse primers used in gene expression analysis

| **Enzyme** | **Gene symbol** | **MSU ID** | **Primer sequences(5'-3')** | **Amplicon size (bp)** |
| --- | --- | --- | --- | --- |
| Nitrate reductase (NR) | *OsNIA1* | LOC_Os02g53130.1 | F-ACTGGTGCTGGTGCTTCTGG  R-CGGCTGGGTGTTGAGGGACT | 104 |
|  | *OsNIA2* | LOC_Os08g36500.1 | F-GAGTCCGACAACTACTACCATT  R-GTTCAGCTCGTTGATGATGTAC | 120 |
|  | *OsNR1* | LOC_Os08g36480.1 | F-AGGCCATGATTTCCCTTTCTAA  R-CCCATGGCTAACAACAATACAG | 97 |
| Nitrate reductase (NiR) | *OsNiR1* | LOC_Os01g25484.1 | 1. GCGATCCTACACCAACCTCC   R-TTCCCTGCAATGCCAACACC | 458 |
|  | *OsNiR2* | LOC_Os01g25520.1 | F-GCCATGAGAAGGTGAAGCTC  R-GTCGGTACTGATGCTTGCG | 177 |
|  | *OsNiR3* | LOC_Os02g52730.1 | F-TGCTCAAGGAGAAGTACCGG  R-CCCATGTCGATGTCCTCCTT | 163 |
| Glutamine synthetase (GS) | *OsGS1;1* | LOC_Os02g50240.1 | F-AGGCACAAGGAGCACATCTC  R-GAAGTAGCCCTTGCCGTTCT | 162 |
|  | *OsGS1;2* | LOC_Os03g12290.1 | F-GAAGTGGAACTACGACGGCT  R-TAGTGGGGATTGGCTCACCT | 161 |
|  | *OsGS1;3* | LOC_Os03g50490.1 | F-ATGTGTGACTGTTATGCG  R-GGTGCTGTAGTTGGTGTG | 492 |
|  | *OsGS2* | LOC_Os04g56400.1 | F-ATCACTTCGCCATGACTTGC  R-GACGTACGGGTCCATGTTTG | 208 |
| Glutamate dehydrogenase (GDH) | *OsGDH1* | LOC_Os03g58040.1 | F-TCTCCGAGCTAGAGCGACTT  R-TCAGTAAGCAGTGAGCGTGG | 499 |
|  | *OsGDH2* | LOC_Os04g45970.1 | F-ATGGGCTGCACGAATCATCC  R-GCACCACCCTCATTTCTGTG | 129 |
|  | *OsGDH3* | LOC_Os02g43470.1 | F-GACATACCTGCTCTCTTG  R-CCTGTTCACCTTCTCTTC | 348 |
|  | *OsGDH4* | LOC_Os01g37760.1 | F-GGTAGCAGTTGGAGAACTCG  R-CACCAACGACTCTGGGTTC | 166 |
| NADP-dependent isocitrate dehydrogenases(NADP-ICDH) | *OsICDH1* | LOC_Os05g49760.1 | F-GGCTTGTTCCAGGATGGACT  R-AACGGATGGACTCGTCAGTG | 210 |
|  | *OsICDH2* | [LOC_Os01g46610](http://rice.plantbiology.msu.edu/cgi-bin/ORF_infopage.cgi?orf=LOC_Os01g46610.1).1 | F-ACCAGAGGACTTGCACACAG  R-TTCCCAGATTCCACGGTTCC | 104 |
|  | *OsICDH3* | LOC_Os01g14580.1 | F-CTGGTGTGCCCTGATGGTAG  R-CGAGCTTTGCCCTATGTCCT | 157 |
|  | *OsICDH4* | LOC_Os04g42920.1 | F-GGTGCTGAACCTGTGGAGTT  R-TGGGCAAGGAAGTCACTCTG | 365 |
| NAD-dependent isocitrate dehydrogenase(NAD-IDH) | *OsIDHc;2* | LOC_Os02g38200.1 | F-CCTTAAGGTGATCACAAAGTTCTGC  R-CCGCCCAAGTCTTTGGTTCT | 549 |
|  | *OsIDHc;1* | LOC_Os04g40310.1 | F-GCATTTGCAGTTCCCGTCAT  R-GGTGCCGCCTAGATCCTTTG | 97 |
|  | *OsIDHa* | LOC_Os01g16900.1 | F-GCTGGTCTGATTGGAGGCTT  R-TCAGGAGCAGAACCGTGAAC | 95 |
|  | *OsIDH1* | LOC_Os03g45320.1 | F-GCCGCAGTTACAGAGACACT  R-GGACTTCCTCTCCCATTCGC | 100 |
| Sucrose-phosphate synthase(SPS) | *OsSPS1* | LOC_Os01g69030.1 | F-TTCAATCCGACGCACTAC  R-CTTCTCGCCTTCAAACAG | 276 |
|  | *OsSPS2* | LOC_Os02g09170.1 | F-TGTTGTCTGGGTCTCTTA  R-TTCCACTGCTCTTCTATC | 214 |
|  | *OsSPS6* | LOC_Os06g43630.1 | F-AGAAGGAGTTGGAGAAGGAGG  R-TGTGTTCCCAGTTGTGCTGT | 173 |
|  | *OsSPS4* | LOC_Os08g20660.1 | F-ATAAAGCGTGGTGTGAGC  R-AGGGTTGGAGAAGAATCG | 186 |
|  | *OsSPS5* | LOC_Os11g12810.1 | F-ATGGGTTGCCGGTAGTGG  R-AGAAGCGGTGGATGTTGC | 186 |
| Trehalose-6-phosphate synthase(TPS) | *OsTPS1* | LOC_Os05g44210.1 | F-AGGAAGGGGATGTGAT  R-CCGAAGAAGGGAATGG | 120 |
|  | *OsTPS4* | LOC_Os03g12360.1 | F-CATCGAGGGATGCAGAGTGG  R-TGGCATGAACCGAAGTCAGG | 178 |
|  | *OsTPS5* | LOC_Os02g54820.1 | F-GTGCTGTCGGAGTTCGTC  R-CCTCATTGCTCGGTGCCT | 418 |
|  | *OsTPS8* | LOC_Os08g34580.1 | F-AGACTGGCAACAGGCTCATC  R-TGTGAAGTAGCCGTGCTCTG | 227 |
|  | *OsTPS9* | LOC_Os09g25890.1 | F-TTGCTCCGACCCTAACAACG  R-AATGAAGTAGCCGTGCTCCG | 118 |
| glutamyl tRNA synthetase(ERS) | *OsERS1* | LOC_Os10g22380.1 | F-ATGACACACCAAAGGAGC  R-TTCACTGGAACGAAGAGC | 320 |
|  | *OsERS2* | LOC_Os01g16520.1 | F-CACCACTTGCCATCCTCTGT  R-GGCGTAGTCGAGCCATTGAT | 224 |
|  | *OsERS3* | LOC_Os02g02860.1 | F-AAGCTGATGGAGTCTGGTGC  R-TTCAACGACCCTTCCTTCGG | 206 |
| Phosphoenolpyruvate carboxylase (PEPCase) | *Osppc1* | LOC_Os02g14770.4 | F-GTAACAGTACAGGGCGAGGT  R-ATTCAACAAACCGTGGCTCC | 214 |
|  | *Osppc2a* | LOC_Os08g27840.1 | F-TCTCAGCCACCAGACACAAT  R-CCACAGCCATCTCATCAAGC | 196 |
|  | *Osppc2b* | LOC_Os09g14670.1 | F-TGTCCGAACTCAACGGGAAG  R-AGAAGAGCCTGGTCAATGCC | 276 |
|  | *Osppc3* | LOC_Os01g55350.1 | F-ACTCAGTCAGTCAGGAGGTC  R-TCATCTGTTCTAAAGGCGGC | 152 |
|  | *Osppc4* | LOC_Os01g11054.1 | F-TCCAGATGGTGTACCGCAAG  R-CTGGAACGTCTCGTCGATGT | 101 |
| glyceraldehyde-3-phosphate dehydrogenase | GAPDH | LOC_Os08g03290.1 | F-GACAGCAGGTCGAGCATCTTC  R-CAGGCGACAAGCTTGACAAAG | 74 |

**Table S2** The gene interaction strengths in these four modules

| Id | Label | degree | modularity_class | CNM |
| --- | --- | --- | --- | --- |
| LOC_Os02g09170.1 | OsSPS2 | 8 | 2 | C |
| LOC_Os08g20660.1 | OsSPS4 | 8 | 2 | C |
| LOC_Os06g43630.1 | OsSPS6 | 8 | 2 | C |
| LOC_Os01g69030.2 | OsSPS1 | 8 | 2 | C |
| LOC_Os11g12810.1 | OsSPS5 | 8 | 2 | C |
| LOC_Os09g25890.1 | OsTPS9 | 13 | 2 | C |
| LOC_Os08g34580.1 | OsTPS8 | 13 | 2 | C |
| LOC_Os05g44210.1 | OsTPS1 | 13 | 2 | C |
| LOC_Os03g12360.1 | OsTPS4 | 13 | 2 | C |
| LOC_Os02g54820.1 | OsTPS5 | 13 | 2 | C |
| LOC_Os01g11054.1 | Osppc4 | 3 | 3 | C |
| LOC_Os09g14670.1 | Osppc2b | 3 | 3 | C |
| LOC_Os02g14770.1 | Osppc1 | 3 | 3 | C |
| LOC_Os01g55350.1 | Osppc3 | 3 | 3 | C |
| LOC_Os08g27840.1 | Osppc2a | 7 | 3 | C |
| LOC_Os02g52730.1 | OsNiR3 | 11 | 3 | N |
| LOC_Os01g25520.1 | OsNiR2 | 11 | 3 | N |
| LOC_Os02g53130.1 | OsNIA1 | 29 | 3 | N |
| LOC_Os08g36480.1 | OsNR1 | 29 | 3 | N |
| LOC_Os08g36500.1 | OsNIA2 | 29 | 3 | N |
| LOC_Os03g45320.1 | OsIDH1 | 11 | 1 | 2OG |
| LOC_Os01g14580.1 | OsICDH3 | 12 | 1 | 2OG |
| LOC_Os01g46610.1 | OsICDH2 | 12 | 1 | 2OG |
| LOC_Os05g49760.1 | OsICDH1 | 12 | 1 | 2OG |
| LOC_Os01g16900.1 | OsIDHa | 13 | 1 | 2OG |
| LOC_Os04g42920.1 | OsICDH4 | 14 | 1 | 2OG |
| LOC_Os02g38200.1 | OsIDHc;2 | 15 | 1 | 2OG |
| LOC_Os04g40310.1 | OsIDHc;1 | 15 | 1 | 2OG |
| LOC_Os04g45970.1 | OsGDH2 | 23 | 1 | 2OG |
| LOC_Os01g37760.1 | OsGDH4 | 23 | 1 | 2OG |
| LOC_Os03g58040.1 | OsGDH1 | 24 | 1 | 2OG |
| LOC_Os01g25484.1 | OsNiR1 | 11 | 4 | N |
| LOC_Os01g16520.1 | OsERS2 | 13 | 4 | C |
| LOC_Os10g22380.1 | OsERS1 | 13 | 4 | C |
| LOC_Os02g02860.1 | OsERS3 | 14 | 4 | C |
| LOC_Os04g56400.1 | OsGS2 | 21 | 4 | N |
| LOC_Os03g12290.1 | OsGS1;2 | 21 | 4 | N |
| LOC_Os02g50240.1 | OsGS1;1 | 22 | 4 | N |
| LOC_Os03g50490.1 | OsGS1;3 | 22 | 4 | N |
| LOC_Os02g43470.1 | OsGDH3 | 22 | 4 | 2OG |

**References**

Ahanger, M.A., Qi, M., Huang, Z., Xu, X., Begum, N., Qin, C., Zhang, C., Ahmad, N., Mustafa, N.S., Ashraf, M., Zhang, L. 2021. Improving growth and photosynthetic performance of drought stressed tomato by application of nano-organic fertilizer involves up-regulation of nitrogen, antioxidant and osmolyte metabolism. Ecotoxicol. Environ. Saf. 216, 112195.

Cao, Y., Fan, X.R., Sun, S.B., Xu, G.H., Hu, J., Shen, Q.R. 2008. Effect of nitrate on activities and transcript levels of nitrate reductase and glutamine synthetase in rice. Pedosphere 18(5), 664-673.

Feng, Y.X., Yu, X.Z., Mo, C.H., Lu, C.J. 2019. Regulation network of sucrose metabolism in response to trivalent and hexavalent chromium in *Oryza sativa*. Journal of agricultural and food chemistry 67(35), 9738-9748.

Galvez, S., Bismuth, E., Sarda, C., Gadal, P. 1994. Purification and characterization of chloroplastic NADP-isocitrate dehydrogenase from mixotrophic tobacco cells. Comparison with the cytosolic isoenzyme. Plant Physiology 105:593–600

Goddijn, O.J., Verwoerd, T.C., Voogd, E., Krutwagen, R.W., De Graff, P.T.H.M., Poels, J., Pen, J. 1997. Inhibition of trehalase activity enhances trehalose accumulation in transgenic plants. Plant physiology 113(1), 181-190.

Hou, W.F., Xue, X.X., Li, X.K., Khan, M.R., Yan, J.Y., Ren, T., Cong, R.H., Lu, J. 2019. Interactive effects of nitrogen and potassium on: grain yield, nitrogen uptake and nitrogen use efficiency of rice in low potassium fertility soil in China. Field Crop Res 236:14–23.

Lin, J., Feng, Y.X., Yu, X.Z. The importance of utilizing nitrate (NO_3_^−^) over ammonium (NH_4_^+^) as nitrogen source during detoxification of exogenous thiocyanate (SCN^-^) in *Oryza sativa*. Environ Sci Pollut Res. [2022](../2022), 29, 5622-5633.

Osuna, L., Gonzalez, M.C., Cejudo, F.J., Vidal, J., Echevarria, C. 1996. In vivo and in vitro phosphorylation of the phosphoenolpyruvate carboxylase from wheat seeds during germination. Plant Physiology 111:551–558

Ratinaud, M.H., Thomes, J.C., Julien, R. 1983. Glutamyl-tRNA synthetases from wheat. Isolation and characterization of three dimeric enzymes. European journal of biochemistry, 135(3), 471-477.

Turano, F.J., Dashner, R., Upadhyaya, A., Caldwell, C.R. 1996. Purification of mitochondrial glutamate dehydrogenase from darkgrown seedlings. Plant Physiology 112:1357–1364
